# Attributing the burden of cancer at work: three areas of concern when examining the example of shift-work

**DOI:** 10.1186/1742-5573-8-4

**Published:** 2011-09-30

**Authors:** Thomas C Erren, Peter Morfeld

**Affiliations:** 1 Institute and Policlinic for Occupational Medicine, Environmental Medicine and Prevention Research, University of Cologne, Germany; 2 Institute for Occupational Epidemiology and Risk Assessment (IERA), Evonik Industries AG, Germany

## Abstract

This commentary intends to instigate discussions about epidemiologic estimates and their interpretation of attributable fractions (AFs) and the burden of disease (BOD) of cancers due to factors at workplaces. By examining recent work that aims to estimate the number of cancers attributable to shift-work in Britain, we suggest that (i) causal, (ii) practical and (iii) methodological areas of concern may deter us from attributable caseload estimations of cancers at this point in time. Regarding (i), such calculations may have to be avoided as long as we lack established causality between shift-work and the development of internal cancers. Regarding (ii), such calculations may have to be avoided as long as we can neither abandon shift-work nor identify personnel that may be unaffected by shift-work factors. Regarding (iii), there are at least four methodological pitfalls which are likely to make AF calculations uninterpretable at this stage. The four pitfalls are: (1) The use of Levin's 1953 formula in case of adjusted relative risks; (2) The use of broad definitions of exposure in calculations of AFs; (3) The non-additivity of AFs across different levels of exposure and covariables; (4) The fact that excess mortality counts are misleading due to the fact that a human being dies exactly once - a death may occur earlier or later, but a death cannot occur more than once nor can it be avoided altogether for any given individual. Overall, causal, practical and methodological areas of concern should be diligently considered when performing and interpreting AF or BOD computations which - at least at the present time - may not be defensible.

## Introduction

Estimates of the burden of disease (BOD) at a population level have been further developed during the last two decades. This has mainly been done with the aim to inform the community and policy makers about the size of a given problem [1]. A world-wide application and an overview of the projects performed were presented in a series of papers. These publications described the World Health Organisation (WHO) programme on the global BOD and comparative risk assessment while focussing on the impact of occupational causes [2-4]. Methodological details were published separately [5]. Recent results regarding the WHO global BOD programme are available on-line [6]. The backbone of all calculations is an estimate of the population attributable fraction (AF) of diseases or deaths (see definition below). Steenland and Armstrong gave a methodological overview on how to calculate AFs and related statistics [7] which has already been commented on [8]. Recently, the BOD methodology was refined and extended by Dr. Rushton and colleagues in order to estimate the cancer burden due to occupational causes in the UK, and to prioritise control measures in an evidence-based manner [9-11]. Moreover, the methodology was used to calculate the cancer burden related to occupational exposures in Europe, extended by a socio-economic assessment and with the aim to amend the EU carcinogen directive [12]. This recent work not only focused on International Agency for Research on Cancer (IARC) Group 1 substances ("established human carcinogens" according IARC categorisation) but it also included IARC Group 2a substances ("probable carcinogens"), and even some Group 2b substances ("possible carcinogens"), such as hydrazine and refractory ceramic fibres.

In brief, Rushton et al. followed and refined the AF procedures as outlined and applied previously [5-7]. First, they identified key epidemiological studies as a basis to estimate a relative risk (RR) for the cancer endpoint of interest. Second, they calculated the prevalence of exposure in a chosen target population by transferring estimates from available (external) sources. Third, they estimated the number and fraction of exposed workers in the target population time-dependently, while taking latency considerations and employment turnover estimates into account. Finally, they calculated AFs and absolute BOD counts by applying formulas that link the RRs taken from the key studies and the derived fractions and numbers of exposed workers (or of exposed cases) in the target population (see below for an explanation of the formulas).

This paper is a commentary on the recent work which aims to estimate the number of cancers attributable to workplace exposures. By examining this strategy of estimating cancer attributable to shift-work in Britain, [10] we have identified a couple of problems that may be involved. Most of our commentary is applicable to other BOD projects as well [1-4,6,12].

## The example of attributing burden of cancer to shift-work

In the course of an expert meeting convened by IARC, scientists came to the conclusion that "shift-work that involves circadian disruption is probably carcinogenic to humans" (Group 2A) [13]. This classification prompted considerable scientific and public interest and numerous - both experimental and epidemiological - studies are underway to settle whether there is a chain of cancer causation or not. Let us suppose that IARC experts were to arrive at a Group 1 classification, i.e., they were to conclude from the available evidence in coming years that there are definite links between shift-work and the development of internal cancers [14]. In such case, we would certainly want to know the impact of such cancer causation in occupational settings. As outlined in the Introduction, tools to arrive to the said quantitative answers by estimating BOD have been developed in the literature. Moreover, the theory and the methodology of such studies have considerably improved in the last decade.

When Rushton et al. "estimated the current burden of cancer in Britain attributable to past occupational exposures for International Agency for Research on Cancer (IARC) group 1 (established) and 2A (probable) carcinogens" in 2010 [10], they included analyses regarding cancer registrations in women which were "attributable" to shift-work. While Dr. Straif, a contributor to the aforementioned IARC classification of shift-work, commended prior work [9] which estimated "attributable fractions and numbers for incidence and mortality" [15], the 2010 calculations came as a surprise to others for several reasons. In 2007, there had already been public discussions, as referred to below, which suggested that such calculations may be uninterpretable.

Clearly, Dr. Rushton and colleagues are to be credited for having taken the lead to combine and analyse large databases in order to attribute incident cancers and cancer deaths to occupational factors. Equally clearly, we think that such work may have to be considered with caution due to concerns in three areas:

(i) Causal area of concern: Is it appropriate to make such calculations when we lack established causality?

(ii) Practical area of concern: Is it appropriate to make such calculations when we lack an effective alternative to shift-work or effective means to intervene to the end of prevention?

(iii) Methodological area of concern: Is it appropriate to make such calculations when their interpretation may be complicated by open methodological questions?

Shift-work, including night work, is widespread and the allegedly linked and attributed cancers of the breast are epidemic. Therefore, attributable fraction and attributable caseload estimations may receive considerable attention and deserve diligent scrutiny.

## Evaluation of concerns

During the Cologne Symposium in 2002 titled "Light, Endocrine Systems and Cancer - Facts and Research Perspectives" [16], possible links between shift-work and breast cancer were one focus of discussion. It should be noted that attributable caseload estimates were already presented [17] and challenged [18] at the symposium. Poole [18] illustrated key pitfalls of such computations in one of four invited critical summaries of the Cologne Symposium:

*Regarding (i): ".... such computations are usually reserved for situations in which a high degree of certainty in the causal hypothesis has accrued. The reason for this caution is that attributable caseload figures are highly newsworthy and exceedingly liable to sensationalisation" *[18].

With regard to (i), this condition is certainly not fulfilled to-date. When the invited IARC experts concluded in October of 2007 that "shift-work that involves circadian disruption is probably carcinogenic to humans" (Group 2A) [13], it came as a surprise to many researchers. Some readers may argue that "probably carcinogenic" already suggests the much-needed high degree of certainty in the causal hypothesis. However, we think that the word "probably" implies a certain degree of uncertainty. Granted, on balance of all available evidence, the IARC experts concluded that there was more evidence for than against the alleged causal links. However, the judgment or anticipation that there is "probably" a causal link is coupled with the knowledge that there may not be. Overall, the available evidence did not suffice to infer causality with the necessary high degree of certainty in October of 2007 [19]. The current IARC Group 2A classification can not be viewed as a one-way street to a Group 1 classification [20] which, in our opinion, could be considered as a necessary requirement for calculations of attributable caseload estimates. By including both proven and probable human carcinogens in their AF analyses, IARC's diligent efforts to judge what causes and what *may *cause cancer could become blurred. Against this background, statements such as "54% of cancer registrations in women are attributable to shift work (breast cancer)" might be considered somewhat premature [10]. Remarkably, according to Table 2 in [10], breast cancer registrations in 2004 attributable to "shift work (including flight personnel)" are estimated to be second in rank, exceeded only by asbestos exposure, among all occupationally "caused" cancers.

*Regarding (ii): "Without a proposed intervention .... attributable fraction and attributable caseload estimates are meaningless". .... "the interest and intent is in eliminating an exposure entirely ...." *[18].

With regard to (ii), attributable caseload computations in [10] are - technically - for a highly implausible, "utopian" society with no shift-work. Alternatives are equally indefensible today and, presumably, in years to come: How could we identify populations of female workers who are 100 percent protected against or robust to the alleged circadian disruption or chronodisruption [19,21,22] that IARC experts expect to be the critical link of a causal chain which "probably" leads to cancer? How should we organise the shift regimens and conditions to become 100 percent innocuous?

Therefore, taken together, the scenario we would want for sensible calculations and interpretation of AFs and attributable caseload estimates regarding shift-work and breast cancer, as dissected by Poole [18] almost a decade ago, may be impossible to defend with today's knowledge.

*Regarding (iii): Are the assumptions for calculations of "attributable fractions and numbers for cancer mortality and incidence using risk estimates from the literature and national data sources to estimate proportions exposed" *[10]*understood and fulfilled?*

With regard to (iii), we - and presumably others - have concerns related to the very methodology used to calculate AFs. At least at this stage, they may not be interpretable. Four possible methodological pitfalls were raised in public by Morfeld at the 19^th ^and 20^th ^International Conferences on Epidemiology in Occupational Health in Banff, Canada, and San José, Costa Rica, respectively (see also [23]): (a) The use of Levin's 1953 [24] formula in case of adjusted relative risks; (b) The use of broad exposure definitions (e.g., a binary exposure indicator) in calculations of AFs; (c) The non-additivity of attributable fractions across different exposures and covariables; (d) The fact that excess mortality figures are misleading because all human beings die exactly once - deaths may occur advanced or may be postponed but no extra deaths or avoided deaths exist.

In the following, we will address methodological pitfalls (1) through (4). We shall do that by examining several theoretical shortcomings. For readers who prefer a non-theoretical refutation of the assertion that the AFs used in [9,10] can be interpreted without ambiguity we shall point to published [(1), (2) and (4)] or provide own [(3)] examples with data sets where such assertions lead to open questions.

## Methodological pitfall (1)

### Levin's formula applied to adjusted risk estimates

The population attributable fraction AF is defined "as the proportion of disease cases over a specified time that would be prevented following elimination of the exposures, assuming the exposures are causal" [25]; p. 15]. To derive expressions for AF we define RR_true _as the true relative risk [26]; counterfactual], RR_crude _as the observed relative risk without any adjustments performed and RR_adj _as the adjusted relative risk after taking confounders into account. Then, AF = pd(RR_true_-1)/RR_true _with pd = proportion of cases in the population exposed to the risk factor. Assuming a successful adjustment of confounders the true relative risk and the adjusted relative risk are identical: RR_true _= RR_adj_. Then AF = pd(RR_adj_-1)/RR_adj _(cp. Formula 4, Table 1 in [25]). This formula is often referred to as "Miettinen's formula" [27]. Under the additional assumption of no confounding, i.e., RR_adj _= RR_crude_, we obtain AF = p(RR_crude_-1)/[p(RR_crude_-1)+1] with p = proportion of exposed subjects in the population (cp. Formula 2, Table 1 in Rockhill et al. 1988 [25]). This formula is often referred to as "Levin's formula" [24]. Note that Levin's formula is guaranteed to be valid only if no confounding occurs. Because epidemiological cancer studies usually adjust relative risk estimates, at least for strong potential confounders like age or sex, the application of Levin's formula in deriving AFs leads to doubtful results in the standard setting. For further discussions see Rockhill et al. [25].

A recent systematic exploration illustrates the inherent problems when applying Levin's formula in AF computations. In 2011 Drs. Darrow and Steenland [28] showed that the use of Levin's 1953 formula [24] in case of adjusted relative risks - as applied in [9,10] in a number of situations - can mislead in significant ways. Darrow and Steenland [28] performed extensive sensitivity analyses under realistic conditions and concluded in their discussion that the observed "bias in the attributable fraction on the order of 20% might be considered substantial, given that estimates of AF are bounded by 0 and 1." Thus, they confirmed the non-negligible potential degree of bias that had already been demonstrated by Greenland [29] in the 1980s (downward bias) and Flegal and co-workers [30] more recently (upward bias). The reader who prefers to be convinced by example is invited to have a look at Table 1 and the Example calculation given by Greenland [29]. He showed that the downward bias in the AF by applying Levin's formula was about 15% in that situation. Note that this kind of bias is expected to occur if any kind of adjustment is performed (because, in that case, confounding is assumed to be non-negligible). Thus, an adjustment of cancer mortality by age suffices - a basic procedure that should have happened in all situations when Levin's formula [24] was applied in [9,10].

## Methodological pitfall (2)

### AF calculation based on a broad definition of exposure

When employing simplified definitions of exposure in [9,10], this use of broad definitions (e.g. binary exposure indicator) in calculations of attributable fractions can be "misleading for projecting the impact of exposure reduction" [31]. Greenland's main argument is obvious and he demonstrated the bias by example. The argument runs as follows: If the exposure distributions among the target and the source populations differ the use of broad definitions of exposure may lead to biased estimates of the attributable fraction (even if the AF is collapsible when analysing the source population). This is so because the result of collapsing data into broad categories depends on the exposure distribution. If the study was from Norway (source) but applied to Britain (target) and exposure distributions differ between the populations of Norway and Britain the collapsed Norwegian effect estimate does not necessarily apply to the British population. Table 1 and Example 2 in [31] gave a worked example with real data from Sweden (source) and the US (target): the biased AF using a dichotomization of exposure was 25%, whereas it was 15% when using three exposure categories. Greenland listed methodological papers that showed how to avoid this error. The author concluded that "it is important to obtain and use detailed exposure and covariate information for attributable fraction estimation" [31]. The authors in [9,10] were probably not able to follow this methodological advice given the data available. They almost always applied broad definitions of exposure: "CAREX was used for estimating the numbers of the British population ever exposed to a carcinogen by industry sector....Industry sectors were ... allocated to 'higher' or 'lower' exposure categories assuming distributions of exposure and risk that corresponded broadly to those of the studies from which the risk estimates were selected" [10]; p. 1429). Furthermore, it is unclear why the word "ever exposed", should have the same meaning in the epidemiological studies (source for estimates of relative risk) and the CAREX exposure system (source for estimates of number of exposed) [32], both applied in the AF estimation process in [9,10].

## Methodological pitfall (3)

### Non-additivity of AFs

This concern pertains to the non-additivity of attributable fractions across different exposures and covariables. Non-additivity can be observed even if the exposures are assumed to be mutually exclusive within the population. To see why let us suppose that there are two exposures and one endpoint (cancer) of interest: Let p_1 _be the prevalence of exposure 1, p_2 _the prevalence of exposure 2 and p_12 _the joint prevalence of exposure 1 and exposure 2. Relative risks associated with these exposures are denoted as RR_1_, RR_2 _and RR_12 _respectively. For the sake of simplicity, let us assume that no confounding exists so that we may rely on Levin's formula [24]. We obtain for the single exposure attributable fractions: AF_1 _= p_1_(RR_1_-1)/(1+p_1_(RR_1_-1)), AF_2 _= p_2_(RR_2_-1)/(1+p_2_(RR_2_-1)). The excess relative risk is ERR = (RR_1_-1)(p_1_-p_12_)+(RR_2_-1)(p_2_-p_12_)+(RR_12_-1)p_12_. Thus the AF_overall _related to both exposures in the population is AF_overall _= ERR/(ERR+1). Example: p_1 _= 0.2, p_2 _= 0.3, RR_1 _= 2, RR_2 _= 3, p_12 _= 0 (mutual exclusive exposures): AF_1 _= 0.167, AF_2 _= 0.375, ERR = 0.8 and AF_overall _= 0.445. However, AF_sum _= AF_1_+AF_2 _= 0.542. Thus, the AFs do not add to AF_overall _even when exposures are mutually exclusive in the population. Note also that the sum of AFs may exceed 1 although there is no synergy. If a multiplicative model is assumed we may try AF_mult _= 1-(1-AF_1_)(1-AF_2_) as an estimate of AF_overall_, see Equation 5 in [5]. When applying this formula to our example we get AF_mult _= 0.479, again a biased estimate in comparison to the true AF_overall _= 0.445. This non-combinability complicates interpretations of AFs when used for planning interventions. In [10] the use of AF_mult _is restricted to joint exposures of the same group (total overlap of exposures) to avoid some of these complications. However, the single AFs depend on the elimination sequence of exposures and confounders in such a population [33]. Therefore, the AF_overall _related to a group of exposures depends on how and when we intervene on (e.g., remove) confounders.

These complications are known. Eide and Gefeller provided a unique solution by introducing average attributable fractions [33,34]. This concept solves the problem of dividing the total proportion of excess cases attributable to the entire set of exposures and covariables into exposure- and covariable-specific components. The attributable excess cases calculated from the average attributable fractions are always additive across all exposures and confounders. Thus, this concept is applicable for solving the problem of shared responsibilities for the occurrence of excess cases in a population. The usual attributable fractions as calculated in [9,10] fail to do so.

## Methodological pitfall (4)

### Excess deaths vs. premature deaths

Excess mortality figures may be misleading because all human beings die exactly once. Deaths may be advanced or may be postponed but, ultimately, there are no extra deaths or avoided deaths. A definition of advanced and postponed deaths in terms of "years of life lost" and "potential years of life lost" is given in [35] applying a counterfactual framework of causality. "The all-to-easy interpretation is that deaths attributed to Factor X will be avoided if exposure to X ceases. Authors may or may not acknowledge explicitly that these are not "avoidable" deaths *per se*, but rather avoidable premature deaths. Death in the end is not avoidable. Death can merely be postponed; what can be influenced is not the fact of death, but its timing" [36], p. 786]. This fact severely restricts the interpretation of body counts.

Consider an ideal cohort study of N subjects, i.e., all N subjects die under observation during follow-up and all causes of death are identified: n_0 _cancer deaths may occur without exposure, but n_1 _given exposure [26] (counterfactual scenario). Let us assume without loss of generality n_1 _> n_0_. This means that the number of non-cancer deaths is N-n_0 _without exposure and N-n_1 _under exposure. Because n_1 _> n_0 _we have N-n_1 _< N-n_0_. This means that exposure is beneficial in terms of body counts when studying non-cancer deaths but detrimental when studying cancer deaths. It follows that the concept of excess deaths calculated from attributable fractions is confusing as an effect measure of the outcome of exposures. This becomes in particular clear when the methodology is applied to all causes of death. If the all cause Standardised Mortality Ratio (SMR) > 1 we expect more deaths under exposure than without exposure when applying the excess death methodology as outlined in [9,10]. However, even without exposure all subjects will die, thus the calculated excess deaths are meaningless: we arrive at the contradiction that more people will die in the exposed cohort than were ever enumerated for study.

One of the problems related to timing may be demonstrated by considering the role of life expectancy in an experimental setting. Consider a medication without side effects that reduces the incidence of heart diseases and, in consequence, heart disease mortality. Without doubt, such an ideal medication is beneficial and protracts life expectancy. If such a medication is given to a cohort still all subjects will die but diseases causing death will potentially change, in particular at higher ages, from heart diseases to other causes, like cancer. When discussing this situation of competing causes of death Lopez wrote [37]; p. 68]: "Given the inevitability of death, a decline in the proportionate mortality from one cause must be compensated by a rise in the proportion of deaths ascribed to others. It is, therefore, probable that persons who previously would have succumbed to one of the cardiovascular diseases (CVDs) are now dying from cancer." Thus, we expect to see, in total, more cancer cases and cancer deaths in a cohort exposed to the medication than in a control cohort unexposed [26]; counterfactual comparison]. We arrive at the paradoxical conclusion that the exposure to this beneficial medication should be restricted because an excess of cancers and cancer deaths is calculated under exposure following the methods used in [9,10]. Thus, an application of these cancer burden methods in deriving and discussing limit values for exposures [11] suffers from potential bias. Lopez identified the reason for this potential bias: "However, what is important is not whether these "saved" persons are dying from cancer (or any other cause) but the *age *at which they are dying from competing causes." [37]; p. 68].

This fundamental problem also affects an informed interpretation of specific excess fractions, e.g., the excess deaths from lung cancer. Greenland [38] presented an example on thyroid cancer in women showing that the problem cannot be overcome by assuming the rare disease assumption. These difficulties are not restricted to risks (cumulative incidence) but also occur when rates are analysed - even when applying sophisticated procedures like Cox modelling techniques [35].

## Conclusions

In their discussion of the example chosen by us to illustrate possible problems which arise when calculating AFs, the authors [10] wrote: "The ramifications of this decision [Denmark recently became the first country to designate breast cancer as an occupational disease eligible for receiving compensation] and of our results could be significant, given the large numbers of women working night shifts in Britain and worldwide". We certainly agree here. We tend to disagree, however, insofar that the aforementioned areas of concern, (i), (ii) and (iii), could serve as possible warning signals with regard to analytical results which - at least at the present time - may be open to several questions.

Overall, that "a significant challenge lying ahead of us worldwide is circadian or chronodisruption and possible causal links with diseases, including cancers" is beyond dispute [13,14,39]. To meet this challenge, and to answer whether shift-work that involves circadian or chronodisruption is causally linked with internal cancers, we need interpretable research. However, at this stage of uncertainty, how should possibly concerned female shift-workers interpret - how should they deal with - statements such as roughly one in two cases of occupationally related breast cancer in women is attributed to shift-work? Beyond the outlined methodological uncertainties, future research may actually show that shift-work involving circadian or chronodisruption does not contribute to cancer. In that case not one female occupationally related breast cancer case would be attributable to shift-work. On the other hand, the BOD attributable to shift-work could be computed to be much higher than "postulated", [10] if research were to demonstrate not only a causal link between shift-work and breast cancer, but also between shift-work and prostate cancer [21,22,39].

To reiterate, it is to the credit of Dr. Rushton and her colleagues for having combined and analysed large databases to attribute incident cancers and cancer deaths to occupational factors. Nevertheless, that combining such diverse databases and complex methodologies may lead to biases should be viewed as the expectation, rather than the exception. Our commentary was intended to point to a number of respective questions. The key task is not to find out whether there may be biases involved but rather to quantify the extent of those biases now. In this vein, we are curious to learn from the authors how much the calculated fractions and case numbers given in [9,10] may be plausibly biased.

Finally, please note that our comments on specific analyses in [9,10] on shift-work and breast cancer are merely intended to exemplify problems which possibly pertain to BOD studies [1,3,6] more generally. In particular, the application of the BOD methodology to calculate the cancer burden related to occupational exposures in Europe, and the extension of these calculations to socio-economic assessments [12] are likely to be affected.

## Competing interests

The authors declare that they have no competing interests.

## Authors' contributions

TCE and PM have conceived of and prepared the material together - with a particular focus by TCE on areas 1 and 2 and by PM on area 3 of concern. Both authors drafted the manuscript and revised it following insightful reviewer comments. TCE and PM have read and approved the final manuscript.
